# Evaluation of Neurotrophic Tyrosine Receptor Kinase 2 (*NTRK2*) as a positional candidate gene for variation in estimated Glomerular Filtration Rate (eGFR) in Mexican American participants of San Antonio Family Heart Study

**DOI:** 10.1186/s12929-015-0123-5

**Published:** 2015-03-25

**Authors:** Farook Thameem, V Saroja Voruganti, John Blangero, Anthony G Comuzzie, Hanna E Abboud

**Affiliations:** Division of Nephrology, Department of Medicine, The University of Texas Health Science Center, 7703 Floyd Curl Drive, San Antonio, TX 78229 USA; Department of Biochemistry, Faculty of Medicine, Kuwait University, Safat, 13110 Kuwait; Department of Nutrition, University of North Carolina at Chapel Hill, Kannapolis, NC 28081 USA; UNC Nutrition Research Institute, University of North Carolina at Chapel Hill, Kannapolis, NC 28081 USA; Department of Genetics, Texas Biomedical Research Institute, San Antonio, TX 78227 USA; South Texas Veterans Healthcare System, San Antonio, TX 78229 USA

**Keywords:** Glomerular filtration rate (GFR), *NTRK2*, Mexican Americans, SOLAR, Genetic variants, SNPs

## Abstract

**Background:**

The estimated glomerular filtration rate (eGFR) is a well-known measure of kidney function and is commonly used for the diagnosis and management of patients with chronic kidney disease. The inter-individual variation in eGFR has significant genetic component. However, the identification of underlying genetic susceptibility variants has been challenging. In an attempt to identify and characterize susceptibility genetic variant(s) we previously identified the strongest evidence for linkage of eGFR occurring on chromosome 9q21 in the Mexican American participants of San Antonio Family Heart Study (SAFHS). The objective of the present study was to examine whether the common genetic variants in Neurotrophic Tyrosine Receptor Kinase 2 (*NTRK2*), a positional candidate gene on 9q21, contribute to variation in eGFR.

**Results:**

Twelve tagging single nucleotide polymorphisms (SNPs) across the *NTRK2* gene region were selected (r2 ≥ 0.80, minor allele frequency of ≥ 0.05) from the Hapmap database. SNPs were genotyped by TaqMan assay in the 848 Mexican American subjects participated in the SAFHS. Association analysis between the genotypes and eGFR (estimated by the Modification of Diet in Renal Disease equation) were performed by measured genotype approach as implemented in the program SOLAR. Of the 12 common genetic variants examined, the rs1036915 (located in 3′UTR) and rs1187274 (located in intron-14), present in perfect linkage disequilibrium, exhibited an association (*P* = 0.017) with eGFR after accounting for the effects of age, sex, diabetes, diabetes duration, systolic blood pressure and blood pressure medication. The carriers of minor allele of rs1036915 (G; 38%) had increased eGFR (104 ± 25 ml/min/1.73 m^2^) in comparison to the carriers of major allele A (98 ± 25 ml/min/1.73 m^2^).

**Conclusion:**

Together, our results suggest for the first time that the genetic variants in *NTRK2* may regulate eGFR.

## Background

Estimated glomerular filtration rate (eGFR) using serum creatinine level is a validated measure of kidney function although creatinine levels are known to be influenced by diet and muscle metabolism. The eGFR is commonly used to identify patients with chronic kidney disease (CKD), a major risk factor for end stage kidney disease. Several studies have reported a strong association between a mild change in eGFR and high risk of cardiovascular death even after accounting for the influences of traditional risk factors such as diabetes, hypertension, and dyslipidemia [1]. Comparing individuals with stable kidney function, both declining and increasing eGFR were independently associated with a higher risk of cardiovascular morbidity and mortality, posing a significant financial burden to the health care systems [2]. Considering the significant health care costs problem associated with CKD and cardiovascular disease, identifying additional risk predictors influencing changes in eGFR overtime is of paramount importance in public health.

Heritability estimate of eGFR demonstrated that 33%-82% of the inter-individual variation in eGFR could be explained by additive genetic effects indicating that changes in eGFR in a given individual is influenced by the genes and their interaction with the environment [3]. Despite intensive research, it has been challenging to identify genetic determinants influencing the complex interplay of inter-individual variation in eGFR. In an effort to identify genes regulating eGFR, we previously performed genome-wide linkage scan in Mexican American participants (N = 848) of the San Antonio Family Heart Study (SAFHS) and identified the strongest evidence for linkage of eGFR to occur on chromosome 9q21 near the markers *D9S301-D9S922* with a LOD score of 3.9 (*P* = 0.00005) [4].

One of the positional candidate genes located within the critical linkage interval is the Neurotrophic Tyrosine Receptor Kinase 2 (*NTRK2*) also known as the Tyrosine receptor kinase B (*TRKB*)/Tropomycin-related kinase B (*TrkB*). NTRK2 belongs to the family of NTRK proteins and is the membrane-bound receptor for the Neurotrophin (NT)-4/5 and Brain-Derived Neurotrophic Factor (BDNF). BDNF binding causes tyrosine phosphorylations in the intracellular domains of NTRK2 which in turn triggers the signaling cascades of mitogen-activated protein kinase (MAPK), phospholipase Cγ (PLCγ), and phosphatidylinositol 3-kinase (PI3K) pathways [5]. Thus, BDNF regulates the development, survival, and differentiation of neurons through binding with NTRK2 [6]. In addition, BDNF/NTRK2 signaling plays a role in human kidney development especially in tubulogenesis and juxtaglomerular apparatus [7,8].

Besides the developmental role, BDNF/NTRK2 signaling appears to be an important downstream target of Melanocortin 4 receptor mediated regulation of food intake and energy expenditure in the hypothalamus [9]. BDNF/NTRK2 signaling increases fat oxidation in skeletal muscle through activation of AMP-activated protein kinase [10]. Furthermore, BDNF/NTRK2 deficiency was associated with increased weight in mice and humans [9,11]. Mutations in *NTRK2* have been associated with obesity and eating disorder in man [12-14].

Given the functional significance of *NTRK2* and its localization on chromosome 9q21, a genetic region linked with eGFR, the objective of the present study is to determine whether the common genetic variants in *NTRK2* are associated with eGFR in the Mexican American participants of SAFHS.

## Methods

### Subjects and phenotypic data

The recruitment of the San Antonio Family Heart Study (SAFHS) family member and data collection procedures from more than 40 extended families have been described previously [15]. Briefly, the cohort was randomly selected from the community with the requirements that they are of Mexican-American ancestry regardless of preexisting medical conditions, part of a large family, and live within the San Antonio region. Blood samples were collected from all participants after an overnight fast and plasma and serum were prepared and stored at −80°C until analyzed. The metabolic, hemodynamic, anthropometric, and demographic variables were collected for all the participants. Diabetes was diagnosed if the 2 h glucose level was 11.1 mmol/l or higher, or if the subject had been prescribed antidiabetic medication. Although a total of 1400 subjects were recruited for SAFHS from 40 large families, kidney-related phenotypic data were collected for only 848 participants from 21 families, who came to the clinic during their 3rd visit. Therefore, this study involves the 848 subjects from 21 families for whom genotypic and phenotypic data are available. GFR was estimated as described previously [4] using the four variables Modification of Diet in Renal Disease (MDRD) formula [16]: *eGFR* (*ml*/min *per* 1.73 *m*^2^) = 186 × [*plasma creatinine* (*mg*/*dl*)] × (*age*)^− 0.203^ × (0.742, *if female*) × (1.210, *if African American*). Estimation of albumin to creatinine ratio (ACR) has previously been described [4]. The quantitative trait values were inverse-normalized and used in the association analyses since their raw data were non-normally distributed. The Institutional Review Board of the University of Texas Health Science Center at San Antonio approved all procedures, and all subjects gave informed consent.

### SNP selection and genotyping

To identify common genetic variations in *NTRK2* gene, tagging SNPs were downloaded from the Hapmap database. The selection of tagging single nucleotide polymorphisms (SNPs) was performed with the tagger program implemented in Haploview (Ver 3.2). Haploview was used to assess linkage disequilibrium for all possible SNP pairs by determining *r*^2^. Twelve tagging SNPs were selected based on the pair-wise tagging (*r*^2^ 0.80, MAF 0.05) using genotype data from the unrelated Hapmap CEU individuals. All the 12 SNPs were genotyped in the study participants (N = 848) by TaqMan assay (Applied Biosystems, CA, USA). Allelic discrimination PCR was carried out on a GeneAmp PCR system 9700 (Applied Biosystems), and fluorescent signals were detected on an ABI PRISM 7700 sequence detector (Applied Biosystems). To assure accuracy of the genotyping, coded blind replicate samples (10%) were included in each assay. Genotypic data with 0% of genotyping error and 0% of inheritance error were subjected to statistical association analyses.

### Statistical association analysis

SNP genotypes were checked for Mendelian consistency using the program SimWalk2 [17]. Allele frequencies were estimated using maximum likelihood estimation methods, which account for the pedigree structure. The estimates of the allele frequencies and their standard errors were obtained using the software package, Sequential Oligogenic Linkage Analysis Routines (SOLAR) [18]. Estimates of linkage disequilibrium (LD) between SNPs were determined by calculating pair-wise D^’^ and r^2^ statistics. Population stratification was tested by the quantitative transmission disequilibrium test (QTDT) as implemented in SOLAR. To investigate the association between *NTRK2* SNPs and eGFR-related traits, the measured genotype analysis (MGA) implemented in SOLAR was employed. This approach extends the classical variance component-based biometrical model to account for both the random effects of kinship and the main effects of SNP genotypes [19]. A p value ≤ 0.05 was considered to be significant. Based on the number of participants (N = 848), there is 83% power to detect an association at the nominal (0.05) level of significance that accounts for as little as 1% of the phenotypic variation.

## Results

Table 1 shows the clinical characteristics of the genotyped individuals. Of the participants, 63% of them were females and the mean age of the study subjects was 48. Among the participants, 22% of them had type 2 diabetes (T2DM). Also, there were about 6% of the participants had eGFR lesser than 60 ml/min/1.73 m^2^.

**Table 1:** **Clinical characteristics of the genotyped SAFHS participants (N = 848)**

**Variables**	**Mean ± SD or %**
Females	63
Type 2 diabetes	21.6
Age (yrs)	47.9 ± 14.8
Systolic blood pressure (mm Hg)	124.4 ± 19.0
Diastolic blood pressure (mm Hg)	69.8 ± 23.0
Body mass index (kg/m^2^)	31.8 ± 7.2
Total Cholesterol (mg/dl)	180.6 ± 38.5
High density lipoprotein-Cholesterol (mg/dl)	48.2 ± 13.6
Triglycerides (mg/dl)	129.2 ± 86.6
Albumin to Creatinine Ratio	0.060 ± 0.4
Estimated Glomerular Filtration Rate (ml/min/1.73 m^2^)	99.2 ± 25.7

**Figure 1:**
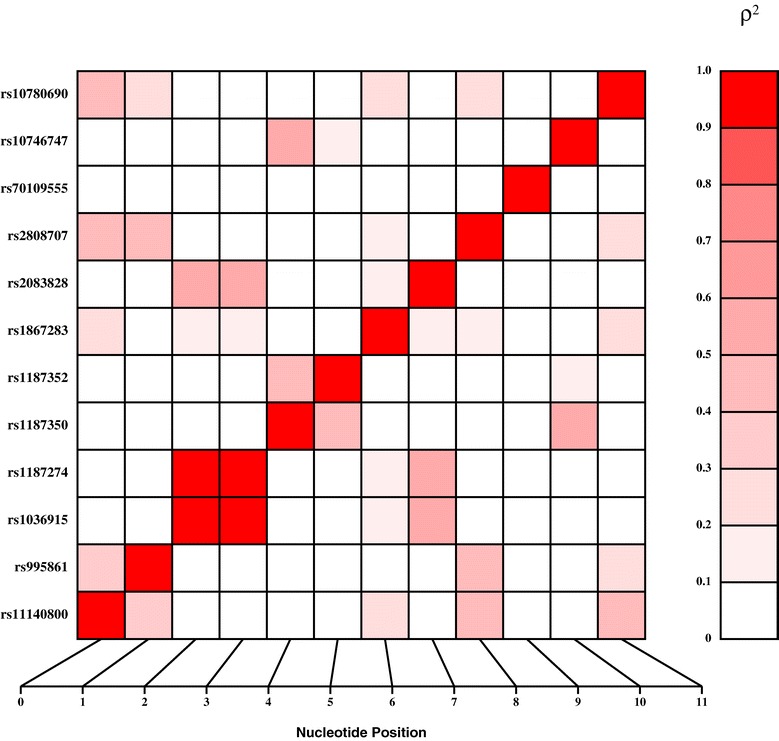
**Linkage disequilibrium (LD) between polymorphisms within the** ***NTRK2*** **gene.** Polymorphisms are labeled on the y-axis. Pairwise LD is estimated using r^2^ values and depicted in the figure by the color intensity of the shaded box. The diagonal represents a comparison of each polymorphism against itself (i.e., r^2^ = 1.0).

Tagging SNPs selected for genotyping in SAFHS participants are shown in Figure 1. Genotypic data of all SNPs tested in our population were consistent with the Hardy-Weinberg Equilibrium expectations. The allele frequencies of the SNPs are shown in Table 2. To identify the hidden population stratification, we performed QTDT that permits a formal test of likely population heterogeneity on a marker-specific basis. Using this test, we found no evidence for such stratification. Furthermore, the QTDT results were consistent with the more powerful measured genotype analyses. Before performing statistical association analysis, we estimated the pairwise LD (r^2^) between all the 12 SNPs and found that the pairwise LD ranged from 0 to 1.0. As can be seen from Figure 1, the high pairwise LD was found for the following SNPs pairs: rs1187274-rs1036915 (r^2^ = 1.0). The rs1187274 and rs1036915 SNPs are located 18 kb away from each other and are in last intron-14 and 3′UTR respectively.

**Table 2:** **Association analysis between the** ***NTRK2*** **polymorphisms and eGFR and SrCr**

**SNPs**	**SNP ID**	**Location on** ***NTRK2***	**Chromosome position (GRCh37/hg19)**	**Major / Minor allele (%) in Europeans (HapMap)**	**Major / Minor allele (%) in Mexican Americans**	**eGFR (** ***P*** values)*	**Serum creatinine (SrCr) (** ***P*** values)*
1	rs11140800	3′UTR	87508137	A(52) / C (48)	A(52) / C (48)	0.184	0.163
2	rs995861	3′UTR	87541642	T (67) / C (33)	T (69) / C (31)	0.298	0.375
3	rs1036915	3′UTR	87437849	A (64) / G (36)	A (62) / G (38)	0.017	0.040
4	rs1187274	Intron-14	87419789	G (58) / C (42)	G (60) / C (40)	0.017	0.038
5	rs1187350	Intron-4	87295237	A (55) / G (45)	A (60) / G (40)	0.408	0.598
6	rs1187352	Intron-4	87293457	C (64) / T (36)	C (78) / T (22)	0.975	0.888
7	rs1867283	3′UTR	87450766	A (51) / G (49)	A (53) / G (47)	0.679	0.521
8	rs2083828	3′UTR	87447045	C (52) / A (48)	C (56) / A (44)	0.126	0.128
9	rs2808707	3′UTR	87558294	G (58) / T (42)	G (54) / T (46)	0.079	0.076
10	rs7019555	3′UTR	87614445	A (71) / G (29)	A (76) / G (24)	0.255	0.551
11	rs10746747	Intron-11	87354637	T (70) / A (30)	T (66) / A (34)	0.960	0.965
12	rs10780690	3′UTR	87478172	G (52) / T (48)	G (54) / T (46)	0.966	0.792

*eGFR (estimated Glomerular filtration rate by MDRD equation) and SrCr (serum creatinine) were adjusted for the effects of age, sex, diabetes, diabetes duration, systolic blood pressure, blood pressure medication.

We performed the association analysis between genotypes and eGFR using measure genotype analysis as implemented in program SOLAR. Table 2 shows the allele frequencies of the SNPs examined and their association with eGFR. Of the 12 SNPs examined for association, the 3′UTR variant rs1036915 (A/G) with minor allele (G) frequency of 38%, exhibited evidence of suggestive association with eGFR (*P* = 0.017) after accounting for the potential covariate effects of the age, sex, diabetes, diabetes duration, systolic blood pressure (SBP), blood pressure medication. The rs1187274 which was in complete LD (r^2^ = 1; Figure 1) with rs1036915 also exhibited a similar association with eGFR. We also performed the association testing using diastolic blood pressure as an additional covariate in our original model and found that the rs1036915 (P = 0.038) and rs1187274 (P = 0.021) were still suggestively associated with eGFR. Since our eGFR linkage region on 9q21 was also linked with serum creatinine (SrCr) in Mexican Americans [4], we extended our investigation whether the *NTRK2* SNPs examined are also associated with SrCr. Interestingly, the two SNPs that showed an association with eGFR were also found to be associated with SrCr levels [rs1036915 (P = 0.038) and rs1187274 (P = 0.040)] after adjusting for the effects of age, sex, diabetes, diabetes duration, systolic blood pressure, and blood pressure medication (Table 2). In addition, we also examined whether *NTRK2* SNPs are associated with other measure of GFR estimated by the Cockcraft-Gault (GC) formula [20], also known as creatinine clearance (CrCl). However, our analysis failed to find an association between the *NTRK2* SNPs and eGFR (estimated by GC equation) after adjusting for effects of age, sex, diabetes, diabetes duration, systolic blood pressure, and blood pressure medication (data not shown).

## Discussion

Heritability of eGFR, which is a measure of how much of the eGFR variability can be attributed to genetic causes and is calculated by observation of phenotypic correlations between relatives in families, is estimated to range from 33% to 82% [3]. Such a vast difference in heritability estimates depends on a number of factors that includes heterogeneous study population, sample size, pedigree structures, ascertainment criteria, treatment effects, definitions of kidney function, diabetes duration, hypertension status, and covariates used to estimate the heritability. For example, Bochud et al. [21], reported a high heritability of eGFR (82%) in a cohort of East African families (348 subjects from 66 pedigrees) each containing at least two hypertensive members. GFR was estimated using Cockcroft-Gault formula. Age and sex were used as covariates in the heritability estimate. We recently reported a heritability of eGFR of 21% in Mexican American families (848 subjects from 26 pedigrees) participated for the SAFHS [4]. SAFHS participants were selected randomly from a census tract in San Antonio of low-income Mexican Americans regardless of any preexisting medical conditions. GFR was estimated by MDRD equation. Heritability was estimated accounting age, sex, BMI, blood pressure medication and diabetic duration as covariates in SAFHS participants. While, the population heterogeneity continues to be a major problem, plus as many factors that may have different genetic regulation influencing eGFR variability, the heritability estimates thus clearly indicate that additive genetic effects have significant influence on the inter-individual variation in eGFR. Family-based genome-wide linkage and association scans are being employed to identify genetic regions where multiple susceptibility genes/genetic variants may aggregate and explain a larger proportion of the heritability of eGFR [4,22-33]. Despite intense research in localization of genes, only a few genes have been associated with eGFR. The susceptibility gene with major effect on eGFR is yet to be identified. In an effort to identify gene(s) regulating eGFR, we previously reported the localization of a genetic region on chromosome 9q21 linked with eGFR in the Mexican participants of SAFHS [4].

The 1-LOD support interval around the linkage signal on 9q21 spans about 15 MB (*D9S301-D9S1120*). A search of current genome databases indicate that there are several expressed sequence tagged sites (ESTs) and approximately 40 genes of known function that have been mapped to this region (https://genome.ucsc.edu). Although none of the known positional genes have been reported to have a direct functional relevance to kidney function, *NTRK2* was selected to screen for SNPs in the present study based on its association with obesity. However, genetic variants in another positional candidate gene, FERM domain containing 3 (*FRMD3*), have been recently reported to be associated with eGFR [32] and diabetic nephropathy (DN) [34]. Although the functional significance of the association of FRMD3 with DN is not clearly known, the promoter variant associated with DN may influence nephropathy through generating the necessary binding site for the proteins involved in the bone morphogenetic protein signaling pathways [35].

In the present study, we scanned the positional candidate gene *NTRK2* for genetic variants to identify whether the common genetic variants in *NTRK2* selected from the HapMap database have any functional relevance to eGFR linkage. Although the HapMap genome browser release #27 merging genotypes and frequencies of “phase 1, 2 and 3” datasets indicates the presence of 39 tagging SNPs in *NTRK2* in unrelated European Caucasians, the 12 tagging SNPs selected and genotyped for the present study were based on the HapMap genome browser release #24; phase 1 in 2008. Furthermore, the LD pattern that we observed in our Mexican American participants (rs1187274-rs1036915; r^2^ = 1.0) was not the same as reported in Europeans, Chinese, Yorubans and Japanese individuals (HapMap release #24; phase 1). Of the 12 common genetic variants selected from the HapMap database and examined for association, the SNP rs1036915 that was in perfect LD with rs1187274 showed a suggestive evidence of an association with eGFR (P = 0.017) after accounting for the effects of confounding factors (Table 2). We tested the mean eGFR values for rs1036915. The carriers of minor allele (G; 38%) had increased eGFR (104 ± 25 ml/min/1.73 m^2^) in comparison to the carriers of major allele A (98 ± 25). Although the functional significance of the association of rs1036915 with eGFR needs to be elucidated, the genetic variants located about 1.5 Mb upstream of rs1036915 have been reported to be associated with eGFR [32] and diabetic nephropathy [34]. Therefore, a thorough high density SNP mapping in and around of the *NTRK2* may identify functional variants that could potentially relate to the GFR linkage finding.

Despite several strengths, our results should be interpreted with caution. Once the issue of multiple testing is accounted, the association observed for rs1036915/rs1187274 with eGFR becomes insignificant. GFR estimated by MDRD equation is not validated in our Mexican American cohorts. However, the GFR estimated using MDRD formula is the best validated method for converting serum creatinine values into eGFR [36] and being used in several linkage studies [22-33]. Although the eGFR levels seem to be normal in our study participants (Table 1), this study was designed to identify the genetic variants influencing variation in eGFR (high or low filtration) in Mexican Americans participants. Also, there were about 6% of participants had eGFR lesser than 60 ml/min/1.73 m^2^.

## Conclusion

In conclusion, we provide evidence for the first time that common genetic variants of *NTRK2* are associated with variation in eGFR although the magnitude of the genetic effects appears to be relatively small. However, the association results presented in this study may be useful for the future meta-analysis validating these SNPs. High density SNP mapping of the critical linkage interval on 9q21 will eventually help to identify the genetic susceptibility variants for eGFR. Identification of susceptibility variants/genes will open a new avenue to elucidate the functional mechanisms by which the variants/genes contribute to renal dysfunction and could eventually help facilitate prediction, development of improved treatment, and prevention of changes in GFR, a strong risk factor for progression of CKD/ESRD as well as associated cardiovascular morbidity and mortality.
